# E4 engages uPAR and enolase-1 and activates urokinase to exert antifibrotic effects

**DOI:** 10.1172/jci.insight.144935

**Published:** 2021-12-22

**Authors:** Shailza Sharma, Tomoya Watanabe, Tetsuya Nishimoto, Takahisa Takihara, Logan Mlakar, Xinh-Xinh Nguyen, Matthew Sanderson, Yunyun Su, Roger A. Chambers, Carol Feghali-Bostwick

**Affiliations:** 1 Division of Rheumatology & Immunology, Department of Medicine, Medical University of South Carolina, Charleston, South Carolina, USA.; 2 Division of Pulmonary Medicine, Department of Medicine, Tokai University School of Medicine, Kanagawa, Japan.

**Keywords:** Pulmonology, Therapeutics, Extracellular matrix, Fibrosis, Rheumatology

## Abstract

Fibroproliferative disorders such as systemic sclerosis (SSc) have no effective therapies and result in significant morbidity and mortality. We recently demonstrated that the C-terminal domain of endostatin, known as E4, prevented and reversed both dermal and pulmonary fibrosis. Our goal was to identify the mechanism by which E4 abrogates fibrosis and its cell surface binding partner(s). Our findings show that E4 activated the urokinase pathway and increased the urokinase plasminogen activator (uPA) to type 1 plasminogen activator inhibitor (PAI-1) ratio. In addition, E4 substantially increased MMP-1 and MMP-3 expression and activity. In vivo, E4 reversed bleomycin induction of PAI-1 and increased uPA activity. In patients with SSc, the uPA/PAI-1 ratio was decreased in both lung tissues and pulmonary fibroblasts compared with normal donors. Proteins bound to biotinylated-E4 were identified as enolase-1 (ENO) and uPA receptor (uPAR). The antifibrotic effects of E4 required uPAR. Further, ENO mediated the fibrotic effects of TGF-β1 and exerted TGF-β1–independent fibrotic effects. Our findings suggest that the antifibrotic effect of E4 is mediated, in part, by regulation of the urokinase pathway and induction of MMP-1 and MMP-3 levels and activity in a uPAR-dependent manner, thus promoting extracellular matrix degradation. Further, our findings identify a moonlighting function for the glycolytic enzyme ENO in fibrosis.

## Graphical abstract

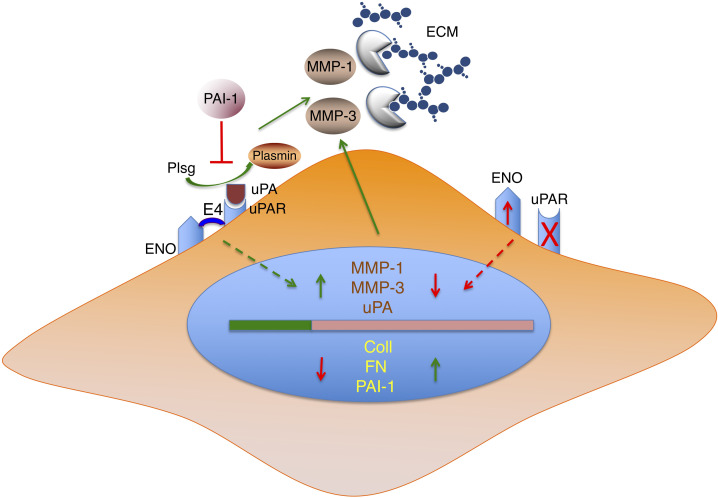

## Introduction

Fibrosis is characterized by fibroblast activation and excess deposition of extracellular matrix (ECM) proteins, such as fibronectin and collagen, in an organ or tissue. Fibrosis results in loss of organ function and is the major cause of morbidity and mortality in progressive fibrotic diseases, such as idiopathic pulmonary fibrosis (IPF) and systemic sclerosis (SSc) (1). SSc is a connective tissue disease of unknown etiology involving the vascular and immune systems and whose hallmark is dermal and organ fibrosis (2, 3). Pulmonary involvement is the leading cause of morbidity and mortality in patients with SSc (3).

There are currently no effective therapies for fibroproliferative disorders such as SSc and IPF. Lung transplantation remains a viable option for a small number of patients. The drugs pirfenidone and nintedanib were approved by the FDA for the treatment of IPF, and the latter was approved for the treatment of SSc (4–6). Although these drugs reduce the rate of disease progression, neither drug halts or reverses lung fibrosis. Thus, the need for the development of an effective therapy for lung fibrosis is undiminished.

We recently demonstrated that the C-terminal domain of endostatin, known as E4, prevents and reverses both dermal and pulmonary fibrosis in vitro, in vivo, and ex vivo (7). Endostatin is the C-terminal region of collagen XVIII. E4 given intratracheally, subcutaneously, and orally ameliorates dermal and pulmonary fibrosis in vivo (7, 8). Furthermore, E4 is effective at ameliorating ongoing fibrosis (7). E4 downregulates a central transcription factor, early growth response protein 1 (Egr-1); decreases collagen and fibronectin transcription; and decreases levels of the ECM cross-linking enzyme lysyl oxidase (7–9). Our goal was to identify cell surface molecules that E4 engages and the pathway by which E4 abrogates fibrosis.

## Results

### Endostatin-derived E4 binds enolase-1 and uPAR.

**Figure 1:**
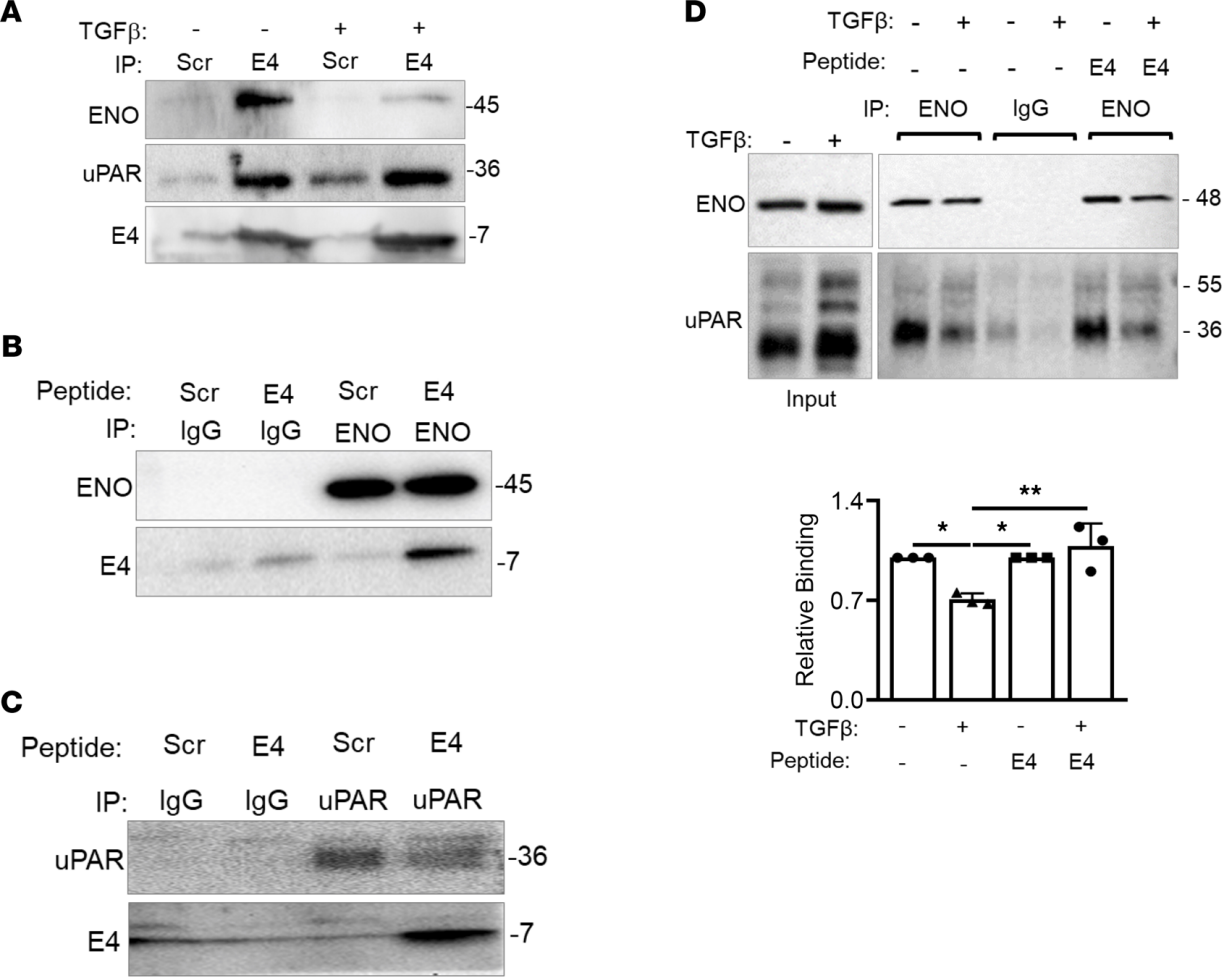
Binding of E4 to ENO and uPAR. (**A**) Cell membrane fractions were extracted from biotinylated-E4 or Scr-treated primary human lung fibroblasts and incubated with neutravidin beads, which bound biotinylated-E4 or Scr. Bound ENO and uPAR were detected using immunoblotting. (**B** and **C**) Cell membrane fractions were extracted from biotinylated-E4 or Scr-treated fibroblasts and incubated with agarose bead–bound anti-ENO antibody (**B**), anti-uPAR antibody (**C**), or isotype control (IgG) (**B** and **C**). Bound E4, ENO, and uPAR were detected using immunoblotting. (**D**) Cell membrane fractions were extracted from control or biotinylated-E4 treated fibroblasts and incubated with agarose bead–bound anti-ENO antibody or isotype control (IgG). Bound uPAR and ENO were detected using immunoblotting. A graphical summary of data from *n* = 3 is shown. Samples were electrophoresed in noncontiguous lanes on the same gel. Statistical analysis was performed using 1-way ANOVA; *P < 0.05, **P < 0.01. Error bars are mean ± SD.

To identify E4 binding partners, we conducted coprecipitation using membrane fractions from primary human lung fibroblasts treated with biotinylated-E4 with or without TGF-β1 and incubated with neutravidin beads. E4-bound proteins were eluted and subjected to mass spectrometry (data not shown). Binding of potential targets was confirmed using precipitation and immunoblotting. Our findings show that independent of TGF-β1 treatment, biotinylated-E4 bound enolase-1 (ENO), a glycolytic enzyme (10), and the urokinase plasminogen activator receptor (uPAR) (Figure 1A). Binding was confirmed using reverse immunoprecipitation. The cell membrane fractions of fibroblasts treated with biotinylated-E4 or scrambled control peptide (Scr) were incubated with anti-ENO antibodies (Figure 1B) or anti-uPAR antibody-immobilized agarose beads (Figure 1C). Bound proteins were identified by immunoblotting. As shown in Figure 1, B and C, E4 pulldown was confirmed using anti-ENO antibodies and anti-uPAR antibodies in fibroblasts treated with E4 but not Scr-treated fibroblasts. We further determined whether ENO and uPAR bound each other. Our data show that ENO bound uPAR at baseline, but the relative binding of these molecules was reduced by TGF-β1; the interaction was restored in the presence of E4 (Figure 1D). Our data suggest that this interaction also occurs in lung epithelial cells (Supplemental Figure 1; supplemental material available online with this article; https://doi.org/10.1172/jci.insight.144935DS1). Thus, our data show that ENO, uPAR, and E4 are binding partners.

### E4 upregulates uPA expression and activity and activates MMP-1 and MMP-3.

**Figure 2:**
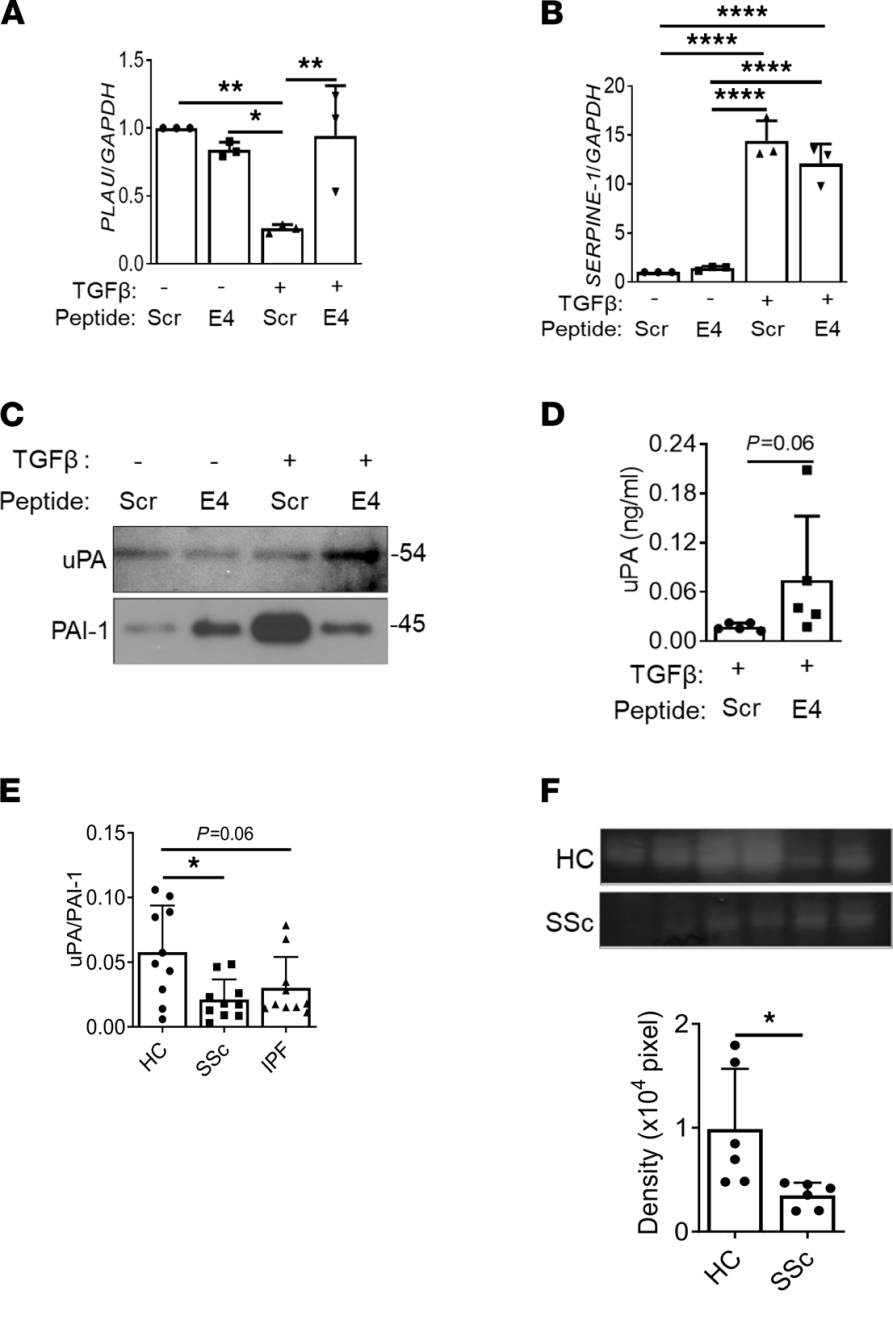
E4 peptide increases uPA levels and decreases in PAI-1 levels in vitro. Normal lung fibroblasts from different donors were treated with TGF-β1 (10 ng/mL) in combination with Scr or E4 peptide for 48 hours. (**A** and **B**) Expression levels of *PLAU* and *SERPINE-1* were measured. (**C**) Protein levels of uPA and PAI-1 were analyzed by immunoblotting of fibroblast culture supernatants. (**D**) Fibroblast culture supernatants treated with TGF-β1 or TGF-β1 in combination with E4 were used for the measurement of uPA activity (*n* = 5/group). (**E**) Lung fibroblasts from HCs (*n* = 12), patients with SSc with PF (*n* = 10), and patients with IPF (*n* = 10) were cultured without any stimulation for 72 hours. The culture supernatants were collected and subjected to activity assays for uPA and PAI-1. The graph shows the ratio of uPA to PAI-1. (**F**) Lung fibroblasts from HCs (*n* = 6) and patients with SSc with PF (*n* = 6) were cultured without any stimulation for 72 hours. The culture supernatants were collected and subjected to casein-plasminogen zymography to detect uPA activity (upper). The graphical summary of casein-plasminogen zymography is shown (lower). Statistical analysis was performed using 1-tailed unpaired Student’s *t* test and 1-way ANOVA as appropriate; *P < 0.05, **P < 0.001, ****P < 0.0001. Error bars are mean ± SD.

ENO moonlights as a plasminogen receptor and enhances plasminogen activation to plasmin by uPAR-bound uPA (10, 11). The plasminogen system comprises plasminogen, tissue plasminogen activator (tPA), uPA, uPAR, and type 1 and 2 plasminogen activator inhibitors 1 and 2 (PAI-1 and PAI-2)(11). The uPA–uPAR–PAI-1 system is known to play an important role in extracellular proteolysis. To investigate whether E4 regulates the plasminogen pathway in vitro, primary human normal lung fibroblasts were treated with TGF-β1 in combination with E4 or Scr, and the effects of E4 on the uPA/plasminogen pathway were examined. TGF-β1 reduced mRNA levels of *PLAU* (the gene encoding uPA) (Figure 2A) and increased those of *SERPINE-1* (the gene encoding PAI-1) (Figure 2B). E4 abrogated these effects and increased the uPA/PAI-1 ratio. Furthermore, this effect was confirmed by immunoblotting of culture supernatants of lung fibroblasts treated with TGF-β1 and E4 (Figure 2C). Measurement of uPA activity revealed that uPA was functionally upregulated by E4 treatment in supernatants of lung fibroblasts (Figure 2D).

To assess the relative levels of uPA and PAI-1 in human fibrotic lung disease, we measured their levels in primary lung fibroblasts derived from patients with SSc who had pulmonary fibrosis (PF) and patients with IPF. The uPA/PAI-1 balance was shifted toward PAI-1 in primary pulmonary fibroblasts from patients with SSc-associated PF and IPF (Figure 2E). Casein-plasminogen zymography showed reduced uPA activity in fibroblasts from patients with SSc-associated PF compared with fibroblasts from healthy controls (HCs) (Figure 2F). These results suggest that the uPA/PAI-I balance is tipped in favor of ECM accumulation, and shifting of this imbalance in favor of uPA is a mechanism by which ECM accumulation and thus, fibrosis, could be reduced.

It is well known that MMPs are central players downstream of uPA and PAI-1. We therefore examined MMP-1 and MMP-3 expression following TGF-β1 and E4 treatment in vitro. TGF-β1 treatment reduced *MMP-1* expression and E4 restored *MMP-1* mRNA levels (Supplemental Figure 2A). E4 also upregulated *MMP-3* expression (Supplemental Figure 2B). Immunoblotting showed that MMP-1 and MMP-3 protein levels were also increased in culture supernatants of normal lung fibroblasts treated with TGF-β1 in combination with E4 (Supplemental Figure 2C). Furthermore, E4 resulted in increased MMP-1 and MMP-3 activity as assessed using collagen and casein zymography, respectively (Supplemental Figure 2D). We also examined the effect of E4 on SSc lung fibroblasts. MMP-1 activity was increased by E4 in SSc lung fibroblasts in a comparable manner to normal lung fibroblasts as assessed by collagen zymography (Supplemental Figure 2E). These findings suggest that E4 ameliorates fibrosis by promoting ECM degradation.

**Figure 3:**
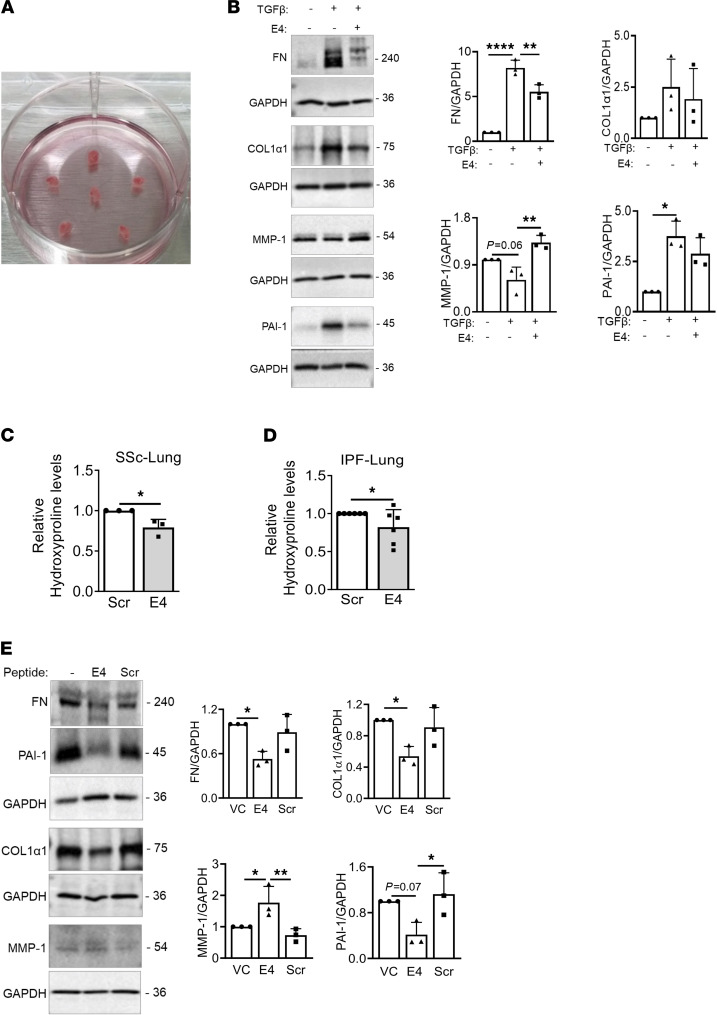
E4 increases MMP-1 and reduces COL1α1, FN, and PAI-1 ex vivo. (**A**) Ex vivo model using human lung tissue cores in organ culture. (**B**) Normal human lung tissues (*n* = 3/group) were treated with TGF-β1 (10 ng/mL) in combination with Scr or E4 peptide (10 μg/mL) for 120–144 hours. Protein levels of FN, COL1α1, MMP-1, and PAI-1 were analyzed by immunoblotting of whole tissue homogenates. Representative Western blots (left) and quantitative analysis (right). (**C** and **D**) SSc (*n* = 3) and IPF (*n* = 6) lung tissue cores were treated with Scr or E4 peptide for 96 hours. The amount of collagen in the lungs was quantified using hydroxyproline assay. (**E**) Lung tissues from patients with SSc (*n* = 3) were treated with Scr or E4 peptide (10 μg/mL) for 72 hours. Representative Western blots (left) and quantitative analysis (right). GAPDH was used as a loading control. Statistical analysis was performed using 1-tailed unpaired Student’s *t* test and 1-way ANOVA as appropriate; *P < 0.05, **P < 0.01, ****P < 0.0001. Error bars are mean ± SD.

To extend our in vitro findings, we conducted ex vivo experiments using human lung tissues in organ culture as we previously described (9, 12, 13). Human lungs from normal organ donors were sliced and 3 mm cores cultured with TGF-β1 in combination with E4 or control peptide as shown in Figure 3A. Immunoblotting of lung core homogenates showed that E4 treatment decreased fibronectin (FN); collagen type I, alpha 1 chain (COL1α1); and PAI-1 protein levels while increasing MMP-1 protein levels (Figure 3B). To confirm the antifibrotic effect of E4 peptide on fibrotic lung tissues, we treated lung tissue cores from SSc and IPF patients with E4 or Scr. E4 significantly reduced the hydroxyproline content of SSc and IPF lungs in organ culture (Figure 3, C and D). In support of these findings, E4 decreased FN, COL1α1, and PAI-1 levels in lung tissue cores from patients with pulmonary fibrosis, while increasing MMP-1 and uPA levels (Figure 3E and Supplemental Figure 3, A–C), demonstrating that E4 can reduce established and end-stage lung fibrosis.

### Plasminogen exerts antifibrotic activity similarly to E4 treatment.

Plasminogen is converted to plasmin by plasminogen activators, and then cell surface–associated plasmin promotes ECM degradation (11). To confirm that plasminogen exerts antifibrotic activity in vitro, normal lung fibroblasts were treated with TGF-β1 with increasing concentrations of plasminogen that are higher than physiologic levels in serum. Plasminogen reduced TGF-β1–induced COL1α1 and FN expression in a dose-dependent manner (Supplemental Figure 4A) while it increased both MMP-1 and MMP-3 activity (Supplemental Figure 4B). Plasminogen was previously reported to induce caspase activation and apoptosis (14). However, plasminogen did not affect caspase-3 levels or cleavage (Supplemental Figure 4C), suggesting that the reduction in ECM was not due to cell apoptosis. The effects of plasminogen were further confirmed by incubating normal lung fibroblasts with TGF-β1 in combination with plasminogen and tranexamic acid (TA), an inhibitor of plasminogen, for 72 hours. Protein levels of TGF-β1–induced collagen expression were reduced by plasminogen, and this effect was abrogated by TA (Supplemental Figure 4D). We next examined the effect of plasminogen on lung fibroblasts of patients with SSc. uPA in combination with plasminogen induced MMP-1 activity in both normal and SSc lung fibroblasts (Supplemental Figure 4E). These results indicate that activation of plasminogen recapitulates the effects of E4, suggesting that plasminogen activation is a plausible mechanism for the antifibrotic effects of E4.

### E4 increases uPA and HGF expression in vivo.

**Figure 4:**
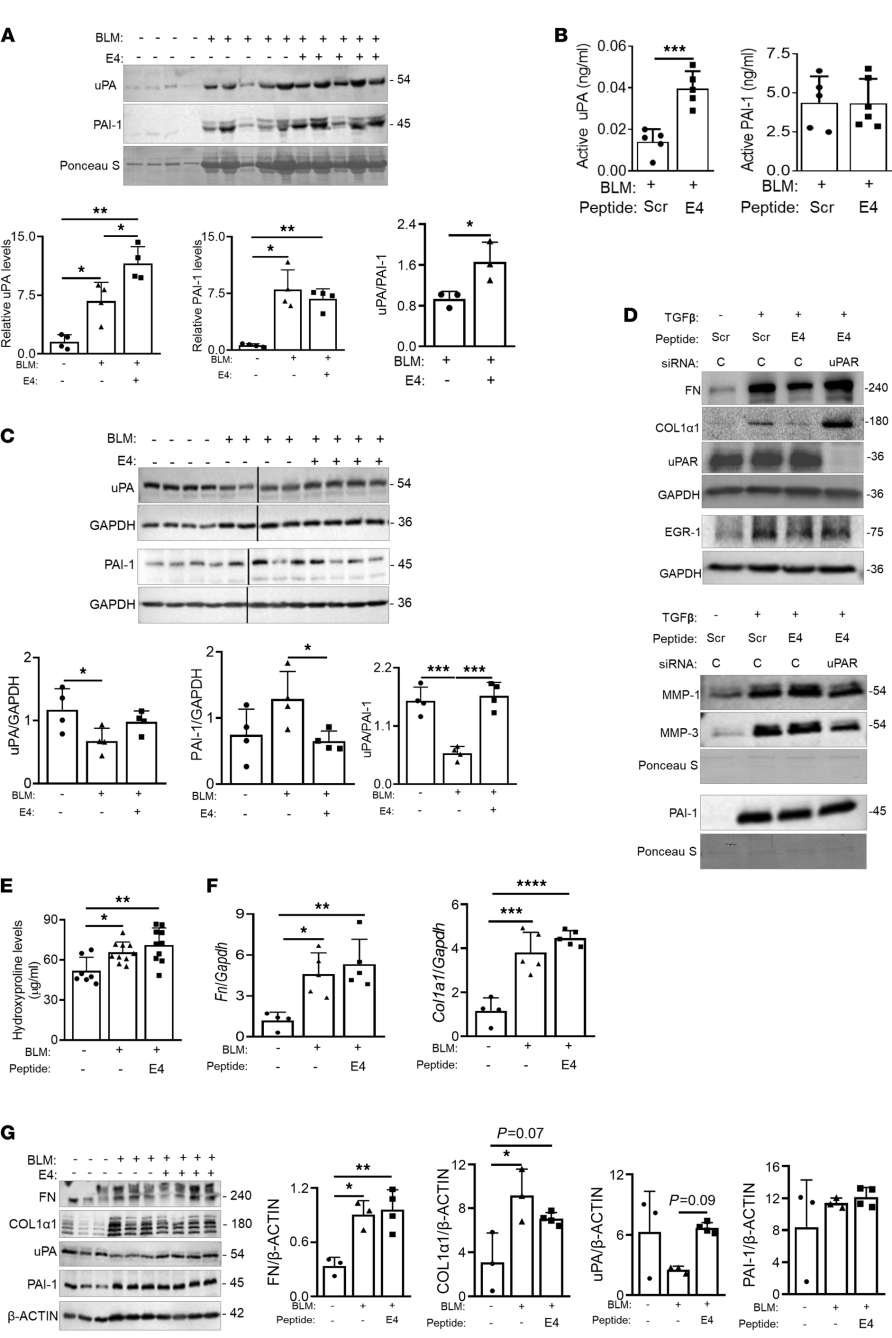
E4 peptide increases uPA and reduces PAI-1 levels in BALF and lung tissues. PBS (*n* = 4), bleomycin (*n* = 5) (1.2 mU/g), or bleomycin and E4 (*n* = 5) (20 μg/mouse) was administered to C57BL/6J male mice. BALF and lung tissues were collected after 14 days. (**A**) Protein levels of uPA and PAI-1 were detected by immunoblotting (upper) and quantitative analysis (lower). (**B**) Activity levels of uPA and PAI-1 in BALF were measured by activity assays. (**C**) Protein levels of uPA and PAI-1 in mouse lung tissue homogenates were detected by immunoblotting. Western blots (upper) and quantitative analysis (lower) are shown. Samples were electrophoresed on the same gel in noncontiguous lanes. Loss of uPAR abrogates the antifibrotic effects of E4. (**D**) Normal lung fibroblasts were transfected with control or uPAR siRNA and treated with Scr or E4 peptide (10 μg/mL) for 72 hours. FN, COL1α1, uPAR, and EGR-1 levels were detected in whole cell lysates (upper), and MMP-1, MMP-3, and PAI-1 were detected in culture media supernatants (lower). GAPDH and Ponceau S stain were used as loading controls for lysates and supernatants, respectively. C, control siRNA. (**E**–**G**) *Plaur^–/–^* mice were treated with PBS (*n* = 7), BLM (*n* = 10) (1.5 mU/g), or BLM with E4 (*n* = 10) (20 μg/mouse). Lung tissues were collected after 14 days. BLM, bleomycin. (**E**) Collagen content quantified by hydroxyproline assay. (**F**) mRNA levels of *Fn* and *Col1a1* were measured relative to the housekeeping gene *Gapdh*. (**G**) FN, Col1α1, uPA, and PAI-1 protein levels in lung homogenates of *Plaur^–/–^* mice detected by immunoblotting. Western blots (left) and graphical presentation of data (right) are shown. β-Actin was used as a loading control. Samples were run in parallel. Statistical analysis was performed using 2-tailed unpaired Student’s *t* test and 1-way ANOVA as appropriate; *P < 0.05, **P < 0.01, ***P < 0.001, ****P < 0.0001. Error bars are mean ± SD.

To extend our in vitro and ex vivo findings, we examined the ability of E4 to increase and activate urokinase in vivo in the bleomycin murine model of pulmonary fibrosis. Bleomycin or PBS was administered intratracheally with or without E4 (20 μg/mouse). Bronchoalveolar lavage fluid (BALF) and lung tissues were collected 14 days after treatment. Protein levels of uPA were increased while those of PAI-1 were decreased in the BALF of mice treated with bleomycin and E4 compared with mice treated with bleomycin and vehicle control, thus increasing the uPA/PAI-1 ratio (Figure 4A). Activity levels of uPA were also significantly increased by E4 treatment while active PAI-1 levels were unaltered (Figure 4B). A similar change was noted in mouse lung tissue homogenates, where bleomycin significantly reduced the uPA/PAI-1 ratio while E4 restored it to levels seen in control mice (Figure 4C).

The release of growth factors, such as HGF, an antifibrotic protein, from the ECM is one of the downstream effects of ECM degradation. We therefore examined whether treatment with E4 alters HGF levels in vivo. Bleomycin reduced *Hgf* expression in mouse lung while E4 significantly increased *Hgf* levels, abrogating the effect of bleomycin (Supplemental Figure 5A). Furthermore, HGF levels increased in BALF of mice treated with bleomycin and E4 (Supplemental Figure 5B). These results indicate that E4 exerts some of its antifibrotic effects via activation of uPA, reduction of PAI-I, and induction of HGF in vivo.

### uPAR silencing increases Col and FN expression.

Since E4 bound uPAR and regulated uPA levels, we next examined the potential role of *uPAR* knockdown on fibrosis-related molecules. Normal lung fibroblasts were transfected with control or *uPAR*-specific siRNA and treated with TGF-β1 in combination with control or E4 peptide. E4 decreased TGF-β1–induced COL1α1 and FN levels, while silencing uPAR abrogated the antifibrotic effects of E4 (Figure 4D). Silencing uPAR also abrogated the E4 induction of MMP-1 and MMP-3 while modestly blocking E4 reduction of PAI-1 (Figure 4D).

To validate these findings in vivo, we treated mice deficient for uPAR (*Plaur^–/–^*) with bleomycin (1.5 mU/g) with and without concomitant E4. Bleomycin increased hydroxyproline levels in mouse lungs (Figure 4E), in agreement with previous findings on the effect of bleomycin in these mice (15). However, E4 did not block the bleomycin effects and thus did not reduce hydroxyproline levels (Figure 4E), in contrast to its effect in WT mice (7, 8). To further confirm this finding, mouse lungs were used to conduct real-time PCR and immunoblotting. E4 did not reduce the expression levels of *Fn* and *Col1a1* in bleomycin-treated mice (Figure 4F). Furthermore, E4 did not reduce the lung tissue levels of COL1α1, FN, or PAI-1 induced by bleomycin, although it restored the bleomycin-induced decrease in uPA levels (Figure 4G). Taken together, our findings show that uPAR is required for E4 to exert its antifibrotic effects but might not be necessary for the E4-mediated increase in uPA, which, in the absence of its receptor, is likely unable to exert its antifibrotic effects.

### Enolase promotes fibrosis.

**Figure 5:**
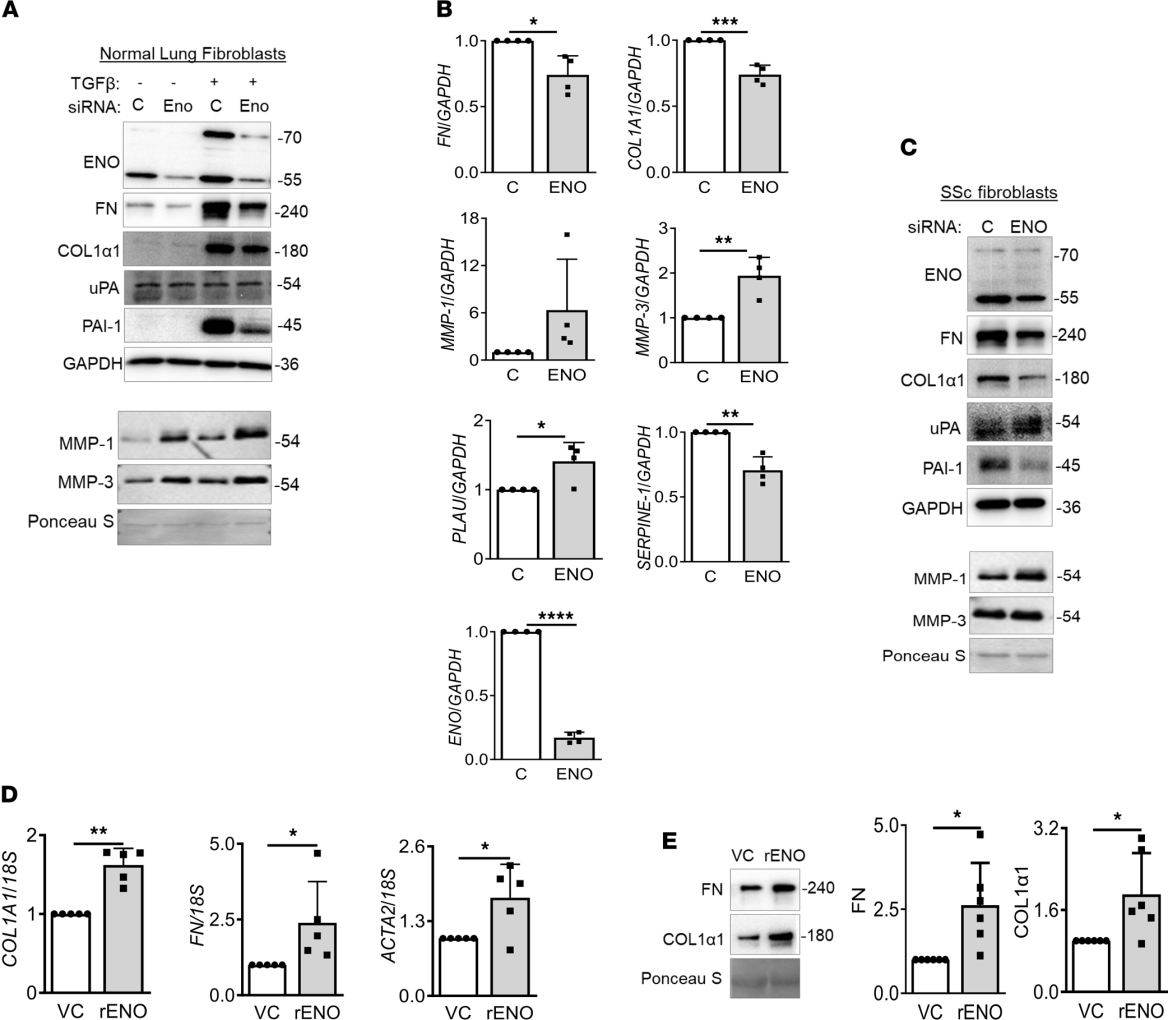
Silencing ENO exerts antifibrotic effects in lung fibroblasts. (**A**) Normal lung fibroblasts were transfected with control or *Eno-1* siRNA, then treated with TGF-β1 (10 ng/mL) for 72 or 96 hours. Expression levels of fibrosis-related genes were measured. Representative Western blots showing protein levels of ENO, COL1α1, FN, uPA, PAI-1, MMP-1, and MMP-3 in lysates and supernatants of lung fibroblasts are shown. (**B**) SSc lung fibroblasts were transfected with control or ENO siRNA for 72 or 96 hours. mRNA expression levels of *ENO*, *COL1A1*, *FN*, *SERPINE-1*, *MMP-1*, *MMP-3*, and *PLAU* were measured relative to the housekeeping gene *GAPDH*. (**C**) Representative Western blots showing protein levels of ENO, COL1α1, FN, uPA, PAI-1, MMP-1, and MMP-3 in lysates and supernatants of SSc lung fibroblasts. GAPDH was used as a loading control. C, control siRNA. (**D**) rENO induces a profibrotic phenotype in vitro independently of TGF-β. MRC-5 were transfected with rENO or control (VC) for 24 hours and 48 hours (*n* = 5). mRNA expression levels of *COL1A1*, *FN*, and *ACTA2* were measured after 24 hours. *18S* was used as a housekeeping gene. (**E**) Protein levels of COL1α1 and FN in supernatants were detected by immunoblotting after 48 hours. Ponceau S stain is used to normalize signals. Representative Western blots (left) and graphical presentation of data (right) (*n* = 6/group). Statistical analysis was performed using 1-tailed unpaired or paired Student’s *t* test as appropriate. *P < 0.05, **P < 0.01, ***P < 0.001, ****P < 0.0001. Error bars are mean ± SD.

The upregulation of the glycolytic pathway due to increased expression of a critical glycolytic enzyme is the early event in myofibroblast differentiation, a hallmark of fibrosis (16). However, the role of the glycolytic enzyme ENO in fibrosis has not been reported to our knowledge. Therefore, we investigated the role of ENO in fibrosis as it interacted with E4 in our assays. Normal lung fibroblasts were transfected with *ENO*-specific siRNA and then stimulated with TGF-β1. mRNA levels of *ENO* were significantly decreased by *ENO* siRNA (Supplemental Figure 6). Protein levels of TGF-β1–induced COL1α1, FN, and PAI-1 were decreased by silencing *ENO*, while those of MMP-1 and MMP-3 increased (Figure 5A). Levels of uPA were not altered following *ENO* knockdown; however, levels of TGF-β1–induced PAI-1 levels were decreased. *ENO* silencing regulated expression of these genes, suggesting that the effect is at the transcriptional level (Supplemental Figure 6). To confirm the effect of *ENO* knockdown on the fibrotic phenotype, SSc lung fibroblasts were transfected with *ENO* siRNA. *ENO* silencing significantly suppressed mRNA and protein levels of COL1α1, FN, and PAI-1 while increasing MMP-1, MMP-3, and uPA (Figure 5, B and C). To determine if ENO can independently promote a fibrotic phenotype, primary lung fibroblasts were treated with recombinant enolase-1 (rENO). rENO significantly upregulated expression levels of the markers of fibrosis *COL1A1*, *FN*, and *ACTA2* within 24 hours (Figure 5D). The increased production of COL1α1 and FN proteins was also detected in supernatants of rENO-transfected fibroblasts 48 hours after transfection (Figure 5E). In parallel, the plasmid encoding an N-terminal V5–tagged ENO was transfected into primary fibroblasts, and the expression was confirmed by Western blot (Supplemental Figure 7A). ENO expression resulted in significant upregulation of *COL1A1* and *FN* mRNA levels and downregulation of *PLAU*, *MMP-1*, and *MMP-3* expression 48 hours after transfection (Supplemental Figure 7B). Similar results were noted at the protein level with increased COL1α1, FN, α–smooth muscle actin (αSMA), and PAI-1 levels in cellular lysates (Supplemental Figure 7C) and decreased MMP-1 and MMP-3 levels in conditioned media (Supplemental Figure 7D) 72 hours and 96 hours after ENO transfection. Protein levels of uPA were unchanged (data not shown).

**Figure 6:**
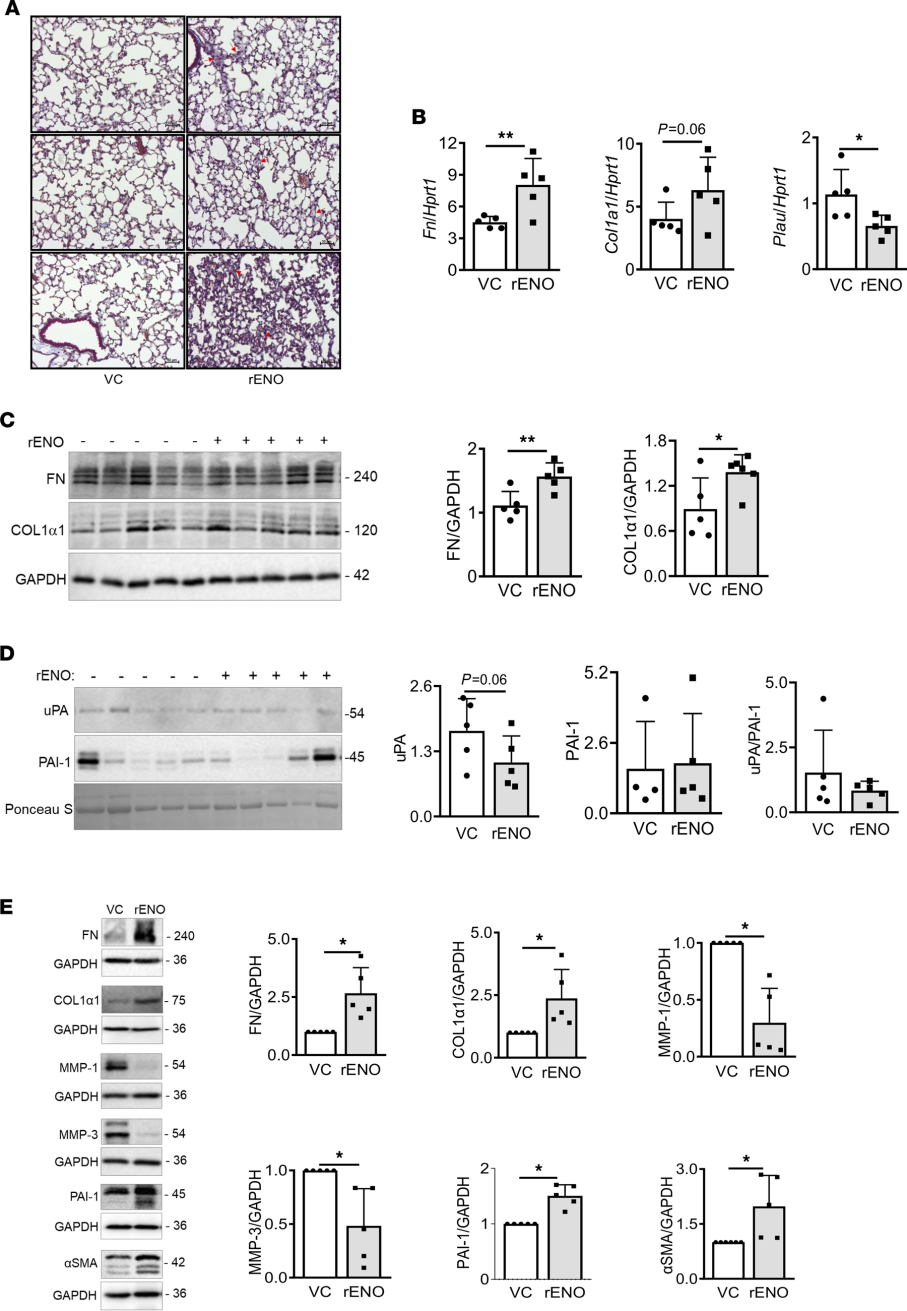
rENO promotes fibrosis in mouse and human lungs. VC or rENO (10 μg/dose) were administered intratracheally to 6- to 8-week-old C57BL/6J mice (*n* = 5/group). Mice were given a total of 4 doses over a period of 2 weeks. BALF and lungs were collected on day 14 after treatment. (**A**) Lung sections of VC- and rENO-treated mice were stained with Masson’s trichrome blue. Scale bars: 50 μm. (**B**) mRNA expression levels of *Fn*, *Col1a1*, and *Plau* were measured and shown relative to the housekeeping gene *Hprt1*. (**C**) Protein levels of FN and COL1α1 in mouse lung homogenates were detected by immunoblotting. Western blots (left) and graphical presentation of the data (right) are shown. GAPDH was used as a loading control. Samples were run in parallel. (**D**) Protein levels of uPA and PAI-1 in BALF of mice treated as above were detected by immunoblotting. Western blots (left) and graphical presentation of uPA and PAI-1 levels and the uPA/PAI-1 ratio (right) are shown. Ponceau S was used to normalize signals. (**E**) Normal human lung tissues (*n* = 5 from 4 independent donors) were treated with VC and rENO protein (4 μg) for 96 hours. Protein levels of FN, COL1α1, MMP-1, MMP-3, PAI-1, and αSMA were detected by immunoblotting. Representative Western blots (left) and graphical presentation of the data (right) are shown. GAPDH was used as a loading control. Statistical analysis was performed using 1-tailed unpaired or paired Student’s *t* test as appropriate; *P < 0.05, **P < 0.01. Error bars are mean ± SD.

Since rENO promoted a fibrotic phenotype in vitro in primary lung fibroblasts, we then examined its effect in vivo. WT C57BL6 mice were given 4 intratracheal doses, 10 μg each, of rENO for 2 weeks. Histological evaluation of lung tissues showed random areas of increased extracellular matrix (Figure 6A). rENO resulted in a significant increase in *Fn* and *Col1a1* mRNA levels in lung tissues while decreasing *Plau* levels (Figure 6B). Similarly, protein levels of FN and COL1α1 in lung tissue homogenates were increased following rENO administration (Figure 6C). In addition, levels of uPA in BALF were decreased while those of PAI-1 were unchanged, resulting in a shift in the uPA/PAI-I ratio (Figure 6D). Finally, to provide direct relevance to the human disease, we treated human lung tissue cores with rENO for 96 hours. Compared with vehicle, rENO significantly increased levels of COL1α1, FN, PAI-1, and αSMA while significantly decreasing levels of MMP-1 and MMP-3 in human lung tissues (Figure 6E). Protein levels of uPA were unchanged (data not shown).

Taken together, our results suggest that E4 engagement of ENO and uPAR is required for the peptide to initiate its antifibrotic cascade, while ENO itself may exert fibrotic effects downstream of TGF-β1, and more importantly, it can independently exert fibrotic effects in vitro, in vivo, and ex vivo.

## Discussion

We have previously shown that E4 is effective in ameliorating fibrosis in multiple complementary preclinical models and tissues including lung and skin (7, 8). However, the specific cell membrane molecules binding E4 and the antifibrotic pathway it activates had not been delineated. In this study, we show that E4 exerts its antifibrotic effects via activation of urokinase and the plasminogen pathway, including activation of MMP-1 and MMP-3, thus promoting ECM degradation. We demonstrate that E4 increases *PLAU* mRNA and protein levels as well as protein activity in vitro and in vivo. Lung-specific inducible uPA expression in transgenic mice protects from bleomycin-induced lung fibrosis (17–19), suggesting that induction of uPA is a viable strategy for reducing fibrosis. Further, gene expression profiles also revealed decreased uPA levels in fibrotic lungs of patients with SSc and IPF (20). Taken together, published findings demonstrate that induction of uPA levels and activity or administration of uPA are effective at reducing fibrosis. In contrast to the increase in uPA, E4 caused a profound decrease in TGF-β1–induced PAI-1 levels. PAI-1 is the primary inhibitor of uPA and tPA and promotes tissue fibrosis by inhibiting uPA-mediated plasmin generation and MMP activation (21). PAI-1–deficient mice treated with bleomycin are protected from pulmonary fibrosis compared with WT mice, while PAI-1–overexpressing mice exhibit more severe fibrosis compared with WT mice (22). In addition to increasing uPA levels and activity directly, E4 enhances uPA activity by decreasing levels of PAI-1, accentuating the antifibrotic activity of E4. These results further lead us to speculate that the balance between uPA and PAI-1 is an important determinant of the extent of fibrosis in tissues. In fact, we showed the lowest uPA/PAI-1 ratio was detected in patients with SSc with pulmonary fibrosis and that E4 increases the uPA/PAI-1 ratio. Moreover, the binding of uPA to its cell surface receptor, uPAR, is required for some of its functions, including ECM degradation (23). The absence of uPAR is associated with the development of dermal and pulmonary fibrosis in mice mimicking human SSc, further emphasizing the importance of the urokinase pathway in fibrosis (24). These findings are supported by our results showing that silencing uPAR in primary human lung fibroblasts abrogates the E4-induced reduction in collagen and fibronectin and loss of uPAR blocks the antifibrotic effects of E4 in vivo. Therefore, E4 engagement of uPAR is required for the peptide to exert its antifibrotic effects. In fact, loss of uPAR in mice prevented E4 from inducing an antifibrotic response, in spite of its increase of uPA levels. This may be due, at least in part, to the fact that uPA bound to uPAR is approximately 200-fold more efficient in activating the cleavage of plasminogen to plasmin compared with free uPA, and thus, the absence of uPAR prevented efficient uPA activity. Although a well-known function of uPAR is promoting plasmin-mediated ECM degradation following the engagement of uPA, uPAR can also engage a variety of cell surface molecules that serve as “coreceptors,” such as integrins, annexin 2, Hsp 70, Hsp 90, thrombospondin, vitronectin, laminin receptor, nucleolin, various G protein–coupled receptors, and receptor tyrosine kinases to promote cell invasion, proliferation, motility, and survival, which can occur independently of uPA engagement and proteolytic activity (25). The loss of uPAR and lack of engagement with 1 or more of these coreceptors may also result in suppression of E4 effects. Although the formation of these uPAR-protein complexes is essential for signaling as uPAR lacks a transmembrane domain, the signaling mechanisms downstream of uPAR are incompletely understood.

In addition to uPA, E4 markedly increased HGF expression in vivo. HGF is known to be sequestered in tissues by binding to ECM components and induces MMP-1 and MMP-3 expression to exert antifibrotic effects (26, 27). HGF also induces expression of uPA and its activity whereas the plasminogen system can enhance HGF effects (28, 29). Our results indicate that E4 can increase uPA and HGF expression, thus participating in a positive feedback loop by which elevated uPA and HGF expression is further increased. Furthermore, HGF can reduce levels of profibrotic factors, such as connective tissue growth factor (30). HGF may contribute to the E4-induced amelioration of fibrosis via increasing MMPs, reducing profibrotic factors, and enhancing the plasminogen system.

ENO is a metalloenzyme (45 kDa) with C-terminal lysines localized to almost all adult tissues (10). It catalyzes the interconversion of 2-phosphoglycerate to phosphoenolpyruvate within glycolysis (31). ENO is expressed on the cell surface, where it acts to promote cell migration and cancer metastasis and participates in systemic infection and the invasion of immune cells through blood vessels and tissues (32). It is also known to serve as a plasminogen receptor and enhances plasminogen activation to plasmin by uPAR-bound uPA (10, 11). This mechanism plays an important role in tumor cell invasion and metastasis (33). Plasminogen binds to its receptors and is subsequently converted to plasmin by plasminogen activators. The cell surface–associated plasmin then facilitates tumor cell invasion by degrading the ECM, promoting cancer development, progression, and spread (33). Indeed, ENO is upregulated in more than 20 types of human cancers, such as gastric cancer, breast cancer, lung cancer, head and neck cancer, and non-Hodgkin’s lymphomas (32). ENO is a therapeutic target in pancreatic ductal adenocarcinomas, where an anti-ENO antibody–based immunotherapy is proposed (34). However, the role of ENO in fibrosis has not been explored. Unlike its mechanism in cancer metastasis, we show that ENO promotes ECM production and reduces MMP-1 and MMP-3 levels and, thus, ECM degradation. We also show that ENO promotes a fibrotic phenotype in vitro, in vivo, and ex vivo in human lung tissues. ENO has 3 isoforms (enolase-1, -2, and -3) with high sequence identity and similar kinetic properties (34). ENO can directly interact with other isoforms to form enzymatically active dimers, as well as with other glycolytic enzymes, such as pyruvate kinase, phosphoglycerate mutase, and aldolase (35). It is likely that E4 binding to ENO serves to sequester this protein, rendering it unavailable for promoting fibrosis. The binding of E4 to ENO may also serve to prevent ENO from binding to plasminogen, thus making plasminogen more available for activation by uPA, which promotes an antifibrotic response. Although this mechanism has not been previously reported, peptide sequestration of profibrotic factors has been reported. For example, a peptide derived from sorting nexin 9 prevents phosphorylated Smad3 from translocating to the nucleus (36).

A correlation between metabolic dysregulation and fibrotic diseases was recently reported (37–40). A recent study showed that TGF-β1 stimulates glycolysis by inducing the accumulation of glycolytic enzyme HK2 in mouse and human lung fibroblasts (41). We now implicate, for the first time, a role for ENO in fibrosis, as expression of ENO in primary fibroblasts upregulated the expression of profibrotic genes and downregulated the expression of MMP-1 and MMP-3, and ENO promoted a fibrotic phenotype in vivo and ex vivo. These findings support a role for ENO in promoting fibrosis independently of TGF-β1.

Our use of human lung tissue cores provides direct relevance of our findings to the human disease. The fact that E4 reduces hydroxyproline levels in end-stage SSc and IPF lung tissues in organ culture further emphasizes the efficacy of E4 at ameliorating fibrosis as these lung tissues represent end-stage disease, and reduction of hydroxyproline levels implies reduction of ECM and fibrosis in advanced lung fibrosis. The reduction in lung fibrosis is complemented by our previous data showing that E4 also ameliorates ongoing dermal fibrosis in mouse and human skin (7) and supports a role for E4 in reversal of fibrosis in 2 different organs relevant for patients with SSc.

In summary, we demonstrated that E4 binds ENO and uPAR. This results in activation of the urokinase pathway with downstream effects, including induction of MMP-1 and MMP-3 levels and activity, thus promoting ECM degradation. E4 also increases uPA and HGF levels, and reduces PAI-1 levels, resulting in further potentiation of the urokinase pathway. The antifibrotic effects of E4 are dependent on uPAR. We further demonstrate that ENO promotes fibrosis in vitro, in vivo, and ex vivo. Taken together, our findings suggest that E4 is a promising therapeutic agent for SSc and other fibrosing diseases that exhibit an imbalance of uPA–PAI-1, and its efficacy is likely due to its ability to activate antifibrotic pathways and suppress profibrotic mechanisms, thus regulating multiple key effectors in fibrosis.

## Methods

Detailed information on the reagents used is provided in Supplemental Table 1.

### Human primary lung fibroblasts.

Lung tissues were obtained from the explanted lungs of patients with SSc and IPF who underwent lung transplantation at the University of Pittsburgh Medical Center and normal controls whose lungs were not used for transplantation as previously reported (20, 42). All tissues were obtained under a protocol approved by the Institutional Review Board of the University of Pittsburgh and the Medical University of South Carolina (MUSC) following written informed consent. Human primary lung fibroblasts were cultured from the explanted lungs of HCs, patients with SSc, and patients with IPF as previously described (42). Briefly, approximately 2 to 3 mm^2^ pieces of tissue were minced, and fibroblasts were cultured and maintained in Dulbecco’s modified Eagle medium (DMEM) (Corning Incorporated Life Sciences) supplemented with 10% FBS (MilliporeSigma), penicillin, streptomycin, and antimycotic agent (Invitrogen). All cells were used between passages 3 and 7.

### Ex vivo human lung culture.

Normal human lung tissues were cut to approximately 2–3 mm^2^, and the pieces of tissue were cultured with TGF-β1 (10 ng/mL) (R&D Systems) in combination with E4 (10–20 μg/mL) in serum-free DMEM supplemented with penicillin, streptomycin, and antimycotic agent. The tissues and media were harvested 120–140 hours following treatment. rENO (4 μg/well) (Abcam) was added to the normal lung tissues and harvested 96 hours after treatment. IPF and SSc lung tissues were cultured with E4 (10–20 μg/mL) or Scr in serum-free DMEM and harvested 72–96 hours after treatment.

### In vivo experiments.

All experiments were done under a protocol approved by the IACUC of the University of Pittsburgh and the MUSC. Pulmonary fibrosis was induced in mice as previously described with some modifications (7). Briefly, bleomycin (1.2 mU/g body weight) in a total volume of 50 μL PBS was intratracheally administrated to 6- to 8-week-old male CB57BL/6J mice (Jackson Laboratory) and uPAR knockout mice (*Plaur^–/–^*), a gift from Victoria Ploplis (University of Notre Dame, Notre Dame, Indiana, USA) (15), at a concentration of 1.2 mU/g and 1.5 mU/g body weight, respectively. E4 (20 μg/mouse) and water as vehicle control were administered via oral gavage concomitantly with bleomycin treatment. Mice were sacrificed by CO_2_ asphyxiation, and BALF and lung tissues were collected 14 days after treatment. In independent experiments, rENO (10 μg) or vehicle control (20 mM Tris-Hcl at pH 7.5, 0.1% glycerol, 1 mM MgSO_4_) were administered intratracheally to mice (CB57BL/6J) every third day (for a total of 4 doses) in a total volume of 100 μL PBS, and lung tissues were harvested after 14 days.

### Bronchoalveolar lavage.

Mice were sacrificed by CO_2_ asphyxiation. The trachea was cannulated and the lungs were lavaged 3 times with 0.7 mL 1× PBS (pH 7.4) containing 0.6 mM EDTA. Lavage fluid was centrifuged to pellet cells and stored at –80°C.

### Immunoprecipitation assay.

A total of 1 × 10^6^ primary human lung fibroblasts were cultured on 100 mm dishes and were either untreated or treated with TGF-β1 for 24 hours. Subsequently, fibroblasts were treated with biotinylated-E4 peptide or biotinylated-Scr for 10 minutes, and the membrane fractions of the cells were extracted using a Subcellular Protein Fractionation Kit (Thermo Fisher Scientific) according to the manufacturer’s protocol. The membrane fractions were incubated with neutravidin beads (Thermo Fisher Scientific) and allowed to bind biotinylated peptide for 4 hours. Bound complexes were subjected to gel electrophoresis. Bound proteins were subjected to mass spectrometry using a commercial service (Bioproximity). As a reverse immunoprecipitation assay, anti-ENO antibody (Abnova), anti-uPAR antibody (R&D Systems), or isotype control was incubated with agarose beads conjugated with protein G (Life Technologies) and mixed with cell membrane fractions extracted from biotinylated-E4 or Scr-treated fibroblasts. For ENO and uPAR binding assays, membrane fractions of fibroblasts treated with TGF-β1 with or without peptide E4 were incubated overnight at 4°C with anti–ENO-1 antibody or isotype control. The protein complex was precipitated using agarose beads conjugated with protein G for 1 hour at 4°C. Immunoprecipitated samples were subjected to SDS-PAGE and immunoblotting as described.

### Cloning and expression of ENO-1.

The human ENO cDNA was amplified by standard PCR from the cDNA of SSc lung fibroblasts using the following primers: *ENO* forward primer (with N-terminal V5 tag and kozak sequence): 5′-TT**GCGGCGCCATG**GGTAAGCCTATCCCTAACCCTCTCCTCGGTCTCGATTCTACGTCTATTCTCAAGATCCA TGC-3′) and *ENO* reverse primer: 5′-CC**CTCGAGCTA**CTTGGCCAAGGGGTTTCTGAAGT-3′) (bold indicates restriction enzyme site and start codon; underline indicates V5 tag sequence). The kozak sequence was added to ensure translation of *ENO*. The cDNA (*ENO*) was subcloned into the mammalian plasmid OG1082 (Oxford Genetics) in the Not1/XhoI restriction site with an N-terminal V5 tag. The positive clones were confirmed by restriction digestion, and all final constructs were sequenced to rule out spurious mutations (Genewiz). To confirm the expression of the positive ENO-1 clones, transfection was done in primary lung fibroblasts using X-tremeGENE Transfection reagent (MilliporeSigma) as per manufacturer’s instructions. Briefly, fibroblasts were seeded at 1.5 × 10^5^ cells per well in 6-well plates (Corning). The culture media were replaced with antibiotic-free media 24 hours after seeding, and fibroblasts were transfected with control (empty vector) or ENO-expressing plasmid. The cells were harvested at 48 hours (for RNA) and 72 hours and 96 hours (for protein) after transfection. To detect tagged ENO protein, fibroblast lysates were subjected to immunoblotting using anti-V5 antibody.

### rENO transfection.

Human fetal lung fibroblasts (MRC-5) were seeded at a density of 1.5 × 10^5^ cells per well in 6-well plates. After 24 hours, rEno (Abcam) was transfected using protein transfection reagent ProJect (Thermo Fisher Scientific). The transfection reagent was prepared as per manufacturer’s instructions. The culture media were replaced with DMEM supplemented with 0.5% FBS (reduced serum media) 1 hour before transfection. For each well, 5 μg rENO protein was added. Tris buffer (20 mM Tris-Hcl at pH 7.5, 1 mM MgSO_4_) was added as vehicle control. The fibroblasts were harvested 24 hours and 48 hours after transfection and processed for RNA and protein extraction, respectively.

### Zymography assay.

Activities of MMPs were examined by zymography as previously described with some modification (43). Briefly, fibroblasts were treated with TGF-β1 in combination with E4 or plasminogen, and the conditioned media were collected for zymography. The conditioned media were separated by electrophoresis on 10% polyacrylamide gels copolymerized with 0.6 mg/mL of rat type I collagen (Corning) for MMP-1 activity or 3 mg/mL of skim milk as a source of casein for MMP-3 activity under nonreducing conditions. After electrophoresis, the gels were washed twice in washing buffer (0.25% Triton X-100 and 50 mM Tris-HCl, pH 7.6) for 30 minutes, incubated with reaction buffer (50 mM Tris-HCl, 10 mM CaCl_2_, 1% Triton X-100, and 0.02% NaN_3_, pH 7.6) for 18 hours at 37°C, and stained with Coomassie blue. In some experiments, 10% polyacrylamide gels copolymerized with 3 mg/mL of skim milk as a source of casein and 18 μg/mL human plasminogen (MilliporeSigma) were used for measurement of uPA activity.

### Activity assay for uPA and PAI-1.

Activities of uPA and PAI-1 in mouse BALF and human lung fibroblast culture supernatants were measured using commercially available kits (Molecular Innovation) following the manufacturer’s instructions.

### ELISA for uPA and PAI-1.

Protein levels of uPA and PAI-1 in human lung fibroblast culture supernatants and protein levels of uPA in lung homogenates were measured using commercially available kits (Molecular Innovation) following the manufacturer’s instructions.

### Hydroxyproline assay.

To quantify the amount of collagen in lung tissues, hydroxyproline content was measured as previously described (44).

### Western blot analysis.

Precipitated materials, fibroblast lysates, conditioned media from fibroblast culture, mouse BALF, and mouse and human lung homogenates were analyzed by immunoblotting as previously described (9). The antibodies used were anti–human ENO-1 antibody (Abcam), anti–human uPAR antibody (MilliporeSigma), anti–human endostatin antibody to detect E4 peptide (R&D Systems), anti–human fibronectin antibody (Santa Cruz Biotechnology), anti–human collagen type I antibody (Abnova, Cedarlane, and CloudClone), anti–human GAPDH antibody (Santa Cruz Biotechnology), anti–human uPA antibody (Proteintech), anti–human PAI-1 antibody (Proteintech), anti–human MMP-1 antibody (Abcam), anti–human MMP-3 antibody (Abcam), anti–human caspase-3 antibody (Cell Signaling Technology), anti–human Egr-1 antibody (Cell Signaling Technology), anti–human β-actin antibody (Santa Cruz Biotechnology), anti–mouse uPA antibody (Abcam), anti–mouse PAI-1 antibody (Proteintech), anti–mouse HGF antibody (R&D Systems), anti–mouse collagen type 1 antibody (Southern Biotech), anti–mouse GAPDH antibody, and anti-V5 antibody (MilliporeSigma) as primary antibodies. Horseradish peroxidase–conjugated antibodies were used as a secondary antibody. Signals were detected using chemiluminescence.

### Quantitative PCR.

Total RNA was extracted from lung tissues and fibroblasts using TRIzol lysis reagent (Life Technologies) and RNeasy Kit (QIAGEN). Reverse transcription was performed with SuperScript IV (Invitrogen). Gene mRNA expression levels were measured by quantitative PCR using the TaqMan real-time PCR system (Life Technologies) according to the manufacturer’s protocol on a TaqMan Gene Expression Assays Step One Plus real-time PCR system (Life Technologies). Each gene’s expression was measured as a ratio to the gene expression of *GAPDH* or *18S* ribosomal RNA (18S). Specific primers and probes for amplifying genes encoding human *PLAU* (Hs01547054_m1), human *SERPINE1* (Hs01126607_g1), human *MMP-1* (Hs00899658_m1), human *MMP-3* (Hs00968305_m1), human *Col1A1* (Hs00164004_m1), human *FN* (Hs00365052_m1), human *ENO* (Hs00361415_m1), human *ACTA2* (Hs00426835_g1), mouse *Fn* (Mm01256744_m1), mouse *Col1a1* (Mm00801666_g1), mouse *Plau*, (Mm01274460_g1), mouse *Hprt1* (Mm03024075_m1), and mouse *Gapdh* (Mm99999915_g1) were purchased from Life Technologies.

### In vitro fibroblast stimulation.

Actively growing primary human lung fibroblasts were stimulated as previously described with some modifications (9). Briefly, 2.0 × 10^5^ primary fibroblasts were plated in 6-well tissue culture plates in 10% FBS-containing DMEM. After 24 hours, the cells were serum-starved in DMEM for 24 hours prior to stimulation with 10 ng/mL of human recombinant TGF-β1 or PBS as vehicle control in combination with 10 μg/mL of biotinylated-E4 or Scr. The cells and conditioned media were harvested 48 hours (for RNA) or 72 hours (for protein) after stimulation. In some experiments, lung fibroblasts were treated with TGF-β1 in combination with plasminogen or plasminogen and tranexamic acid instead of E4 peptide.

### siRNA transfection.

Primary human lung fibroblasts were seeded at a density of 2 × 10^5^ cells per well in 6-well plates 24–48 hours prior to transfection with siRNA. uPAR-specific siRNA and control siRNA were purchased from Life Technologies. ON-TARGETplus Eno-1–specific siRNA and ON-TARGETplus control siRNA were purchased from Dharmacon. Transfection was done using Lipofectamine 2000 (Invitrogen), and 100 nmol siRNA was diluted in Opti-MEM I Reduced-Serum Medium (Life Technologies) following the manufacturer’s recommendation. TGF-β1 was added to media of cells 24 hours after transfection. Fibroblasts were harvested 48 hours (for RNA) and 72 hours (for protein) after treatment.

### Histological analysis.

SSc lung tissues treated with E4 peptide were harvested, fixed in 10% formalin, and embedded in paraffin. Paraffin-embedded tissue sections of 5 μm were processed for H&E staining (Histology & Immunohistochemistry Laboratory, MUSC). Masson’s trichrome staining was performed for mouse lungs treated with rENO protein and VC (AML Laboratories). Images were captured on Axio Observer Microscope (Carl Zeiss Microscopy).

### Statistics.

All continuous variables were expressed as the mean ± standard deviation. All statistical analyses were done using GraphPad Prism 8.00 for Windows (GraphPad Software). Comparisons between 2 groups were tested for statistical significance with the unpaired/paired 1- or 2-tailed Student’s *t* test, as appropriate. Comparison among 3 or more groups was performed using 1-way ANOVA followed by Tukey’s multiple-comparison test.

### Study approval.

The use of mice was approved by the Institutional Animal Care and Use Committee of the Medical University of South Carolina. The use of human lung tissues was approved by the Institutional Review Boards of the University of Pittsburgh and the MUSC.

## Author contributions

TN, TW, TT, SS, and CFB designed the experiments. TW, TN, and SS performed the research; collected, analyzed, and interpreted data; and wrote the manuscript. TT, LM, XXN, MS, YS, and RAC performed the research and collected data. TW, TN, SS, and CFB interpreted data and contributed to manuscript writing. CFB designed the research, interpreted data, supervised and organized the study, and wrote and edited the manuscript. All authors edited and approved the final version. TW and SS contributed as co–first authors, and authorship order is based on the amount of data contributed.

## Supplementary Material

Supplemental data

## Version 1 12/22/2021

Electronic publication
