# Supplementary material for: E4 engages uPAR and enolase-1 and activates urokinase to exert antifibrotic effects
Source: JCI Insight. 2021 Dec 22;6(24):e144935. doi: 10.1172/jci.insight.144935 (PMC8783693; doi:10.1172/jci.insight.144935)
Supplement: Supplemental data [file jciinsight-6-144935-s087.pdf]

Supplemental Table 1: Materials

REAGENT or RESOURCE	SOURCE	CATALOG #
**Antibodies**		
β-actin (C4)	Santa Cruz Biotech	Sc-47778
Caspase-3	Cell Signaling Technology	9662
Collagen type 1α1	Abnova	PAB17204
Collagen type 1α1	CloudClone	PAA350Hu01
Collagen type 1α1	Cedarlane Labs	CL7812AP-1
Collagen type 1α1	Southern Biotech	1310-01
EGR1 (15F7)	Cell Signaling	4153
ENO1 {EPR10864(B)}	Abcam	Ab155955
ENO1(8G8)	Abnova	H00002023-M01
Endostatin	R&D Systems	AF1098
Fibronectin (EP5)	Santa Cruz Biotech	8422
GAPDH (0411)	Santa Cruz Biotech	SC-47724
GAPDH (EPR16891)	Abcam	ab201822
HGF	R&D Systems	AF-294-NA
MMP-1 (EP1249Y)	Abcam	ab134184
MMP-3 (EP1186Y)	Abcam	ab52915
PAI-1	Proteintech	13801-1-AP
PAI-1 (1H4A5)	Proteintech	66261-1-Ig
uPA	Proteintech	17968-1-AP
uPA	Sigma-Aldrich	SAB1105036
uPAR (62022)	R&D Systems	MAB807
uPAR	Sigma-Aldrich	SAB2700745
V5	Sigma-Aldrich	V8012
Mouse IgG-HRP	Promega	W402B
Rabbit IgG-HRP	Amersham	NA934V
Goat IgG-HRP	Santa Cruz Biotech	sc-2354
Streptavidin-HRP	Amersham	RPN1231
**Bacterial and Virus Strains**		
None		
**Restriction Enzymes**		
NotI-HF	New England Biolabs	R1389
XhoI	New England Biolabs	R0146
**Biological Samples**		
Normal lung tissues	University of Pittsburgh Medical Center (UPMC)/ Medical University of South Carolina (MUSC)	N/A
SSc lung tissues	UPMC/MUSC	N/A
IPF lung tissues	UPMC/MUSC	N/A

Supplemental Table 1: Materials

**Reagents, Peptides, and Recombinant Proteins**		
Dulbecco's modified Eagle medium	Corning Incorporated Life Sciences	10-013-CV
Fetal bovine serum	Sigma-Aldrich	F4135
Penicillin-Streptomycin	Invitrogen	15140122
E4	Peptide Synthesis Facility (University of Pittsburgh)	N/A
Biotinylated-E4	Peptide Synthesis Facility (University of Pittsburgh)	N/A
Bleomycin	Hospira Inc	61703-332-18
Neutravidin-beads	Thermo Fisher scientific	29200
Agarose beads	Life Technologies	15920-010
X-tremeGENE Transfection reagent	Millipore Sigma	XTG9-RO
Protein transfection reagent ProJect	Thermo Fisher scientific	89850
Rat type I collagen	Corning	354236
Active uPA protein	Abcam	Ab167714
Human TGF-β	R&D Systems	100-B
Human plasminogen	Sigma-Aldrich	P7999-5UN
Recombinant Eno1	Abcam	ab89248
TRIZOL Lysis Reagent	Life technologies	15596026
Lipofectamine 2000	Invitrogen	11668019
Opti-MEM I	Life Technologies	31985062
Taqman Universal PCR master mix	Thermo Fisher scientific	43-044-37
RNeasy® kit	Qiagen Inc	74106
SuperScript IV	Invitrogen	18090050
**ELISA Kits**		
Subcellular Protein Fractionation Kit	Thermo Fisher scientific	78840
Human uPA total antigen assay ELISA kit	Molecular Innovations	HUPAKT-TOT
Active human uPA functional assay ELISA kit	Molecular Innovations	HUPAKT
Active mouse uPA functional assay ELISA kit	Molecular Innovations	MUPAKT
Active human PAI-1 functional assay ELISA kit	Molecular Innovations	HPAIKT
Active mouse PAI-1 functional assay ELISA kit	Molecular Innovations	MPAIKT
**Experimental Models: Cell Lines**		

Supplemental Table 1: Materials

Human fetal lung fibroblasts (MRC5)	ATCC	CCL-171
Human alveolar epithelial cells (A549)	ATCC	CCL-185
**Experimental Models: Organisms/Strains**		
C57BL/6J mice	The Jackson Laboratory	000664
uPAR KO *(Plaur -/-)* Mice (C57BL/6J)	W.M. Keck Center for Transgene Research, Notre Dame, IN	
**Oligonucleotides**		
The human Enolase-1 cDNA forward primer:5'TTGCGGCGCCATGGGTAAGCCTATCCCTAACCCTCTCCTCGGTCTCGATTCTACGTCTATTCTCAAGATCCA TGC-3'	Integrated DNA Technologies	N/A
The human Enolase-1 cDNA reverse primer: 5'-CCC TCG AGC TAC TTG GCC AAG GGG TTT CTG AAG T-3'	Integrated DNA Technologies	N/A
uPAR-specific siRNA	Life Technologies	4390824
ON-TARGETplus Eno-1-specific siRNA	Dharmacon	J-004034-05-0050
**Taqman Real Time PCR Systems**		
ACTA2	Life Technologies	Hs00426835_g1
COL1A1	Life Technologies	Hs00164004_m1
COL1A1	Life Technologies	Mm00801666_g1
COL1A2	Life Technologies	Hs00164099_m1
Eno1	Life Technologies	Hs00361415_m1
FN1	Life Technologies	Hs00365052_m1
FN1	Life Technologies	Mm01256744_M1
GAPDH	Life Technologies	Hs02758991_g1
GAPDH	Life Technologies	Mm99999915_g1
HPRT1	Life Technologies	Mm03024075_m1
MMP-1	Life Technologies	Hs00899658_m1
MMP-3	Life Technologies	Hs00968305_m1
PAI-1/Serpine1	Life Technologies	Hs01126607_g1
uPA/Plau	Life Technologies	Hs01547054_m1
uPA/Plau	Life Technologies	Mm01274460_g1
18S	Life Technologies	Hs_99999901_s1
**Recombinant DNA**		
Mammalian plasmid OG1082	Oxford Genetics	OG1082

Supplemental Table 1: Materials

**Software and Algorithms**		
GraphPad Prism 8.00/9.00 software	GraphPad Prism Software, Inc	N/A

Supplemental Figures and Figure Legends

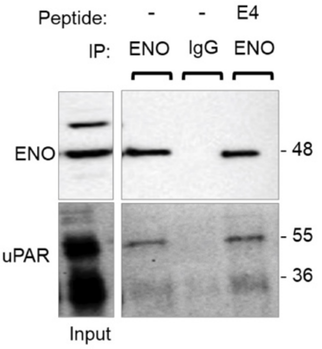

Supplemental Figure 1. ENO binds uPAR in human lung epithelial cells. Cell membrane fractions were extracted from control or Biotinylated E4-treated lung epithelial cells (A549) and incubated with agarose beads-bound anti-ENO antibody or IgG isotype control (IgG). Bound uPAR and ENO were detected using immunoblotting. Total cell membrane fractions are included as input. The samples were run on the same gel but were in noncontiguous lanes.

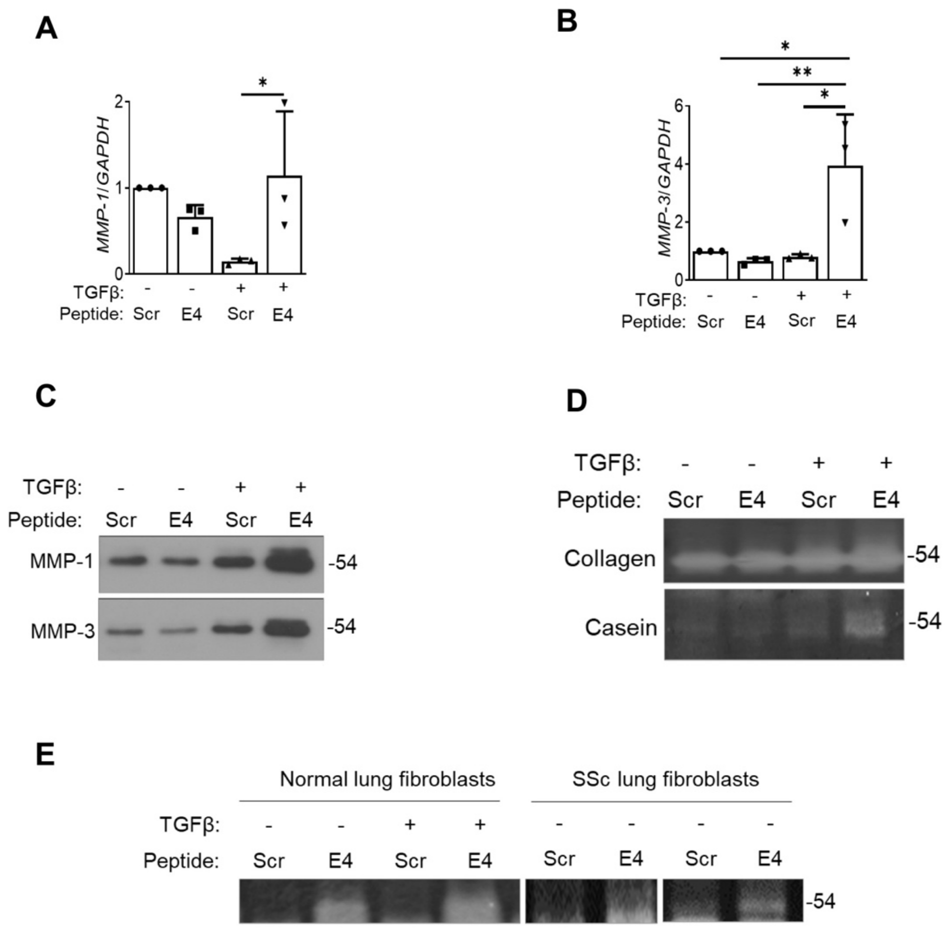

Supplemental Figure 2. E4 peptide increases the expression and activity of MMP-1 and MMP-3 in vitro. Normal lung fibroblasts were treated with TGF-β1 (10 ng/ml) in combination with scrambled (Scr) or E4 peptide (10 µg/ml) for 48 and 72 hours. (A) Expression levels of *MMP-1* and (B) *MMP-3* mRNA were measured. (C) Protein levels of MMP-1 and MMP-3 were analyzed by immunoblotting of fibroblast culture supernatants. (D) Representative data from zymography are shown. Fibroblast culture supernatants treated with TGF-β1 in combination with E4 for 72 hours were used for detection of MMP activity. Substrates used in zymography are collagen type I (for MMP-1 activity) and casein (for MMP-3 activity). (E) Normal and SSc lung fibroblasts were treated with scrambled (Scr) or E4 peptide for 72 hours. Representative data from zymography are shown. Fibroblast culture supernatants were used for detection of MMP activity by zymography using collagen type I as substrate (for MMP-1 activity). Statistical analysis was performed using unpaired Student's t-test $*p < 0.05$, $** p < 0.01$. Error bars are mean +/- SD.

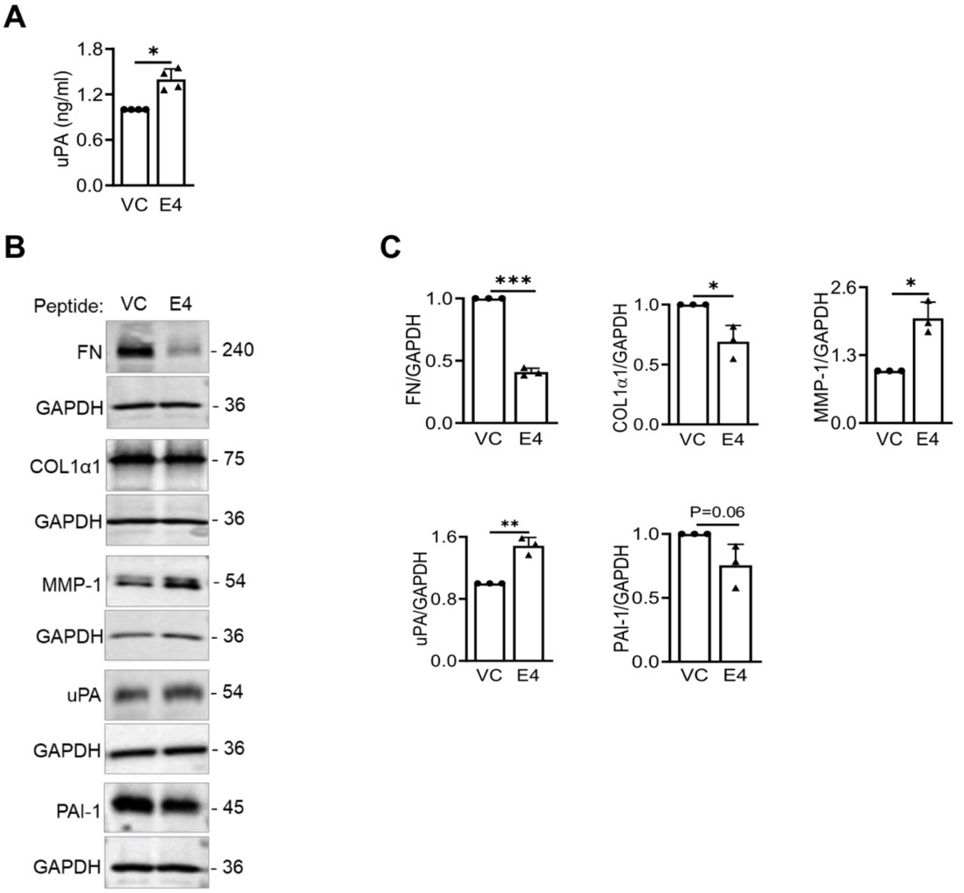

Supplemental Figure 3. E4 exerts anti-fibrotic effects in human lung tissues. (A) Lung tissues from SSc patients (N=4) were treated with control or E4 peptide (10 μg/ml) for 72 hours. uPA protein levels in lung homogenates were measured by ELISA. (B) Lung tissues from IPF patients (N=3) were treated with control or E4 peptide (10μg/ml) for 72-96 hours. (B) Protein levels of FN, COL1α1, MMP-1, uPA and PAI-1 in lung homogenates were detected by immunoblotting. GAPDH was used as a loading control. Representative western blots (Left) and graphical presentation of the data (Right) are shown. Statistical analysis was performed using paired Student's t-test. $*p < 0.05$, $** p < 0.01$, $*** p < 0.001$. Error bars are mean +/- SD.

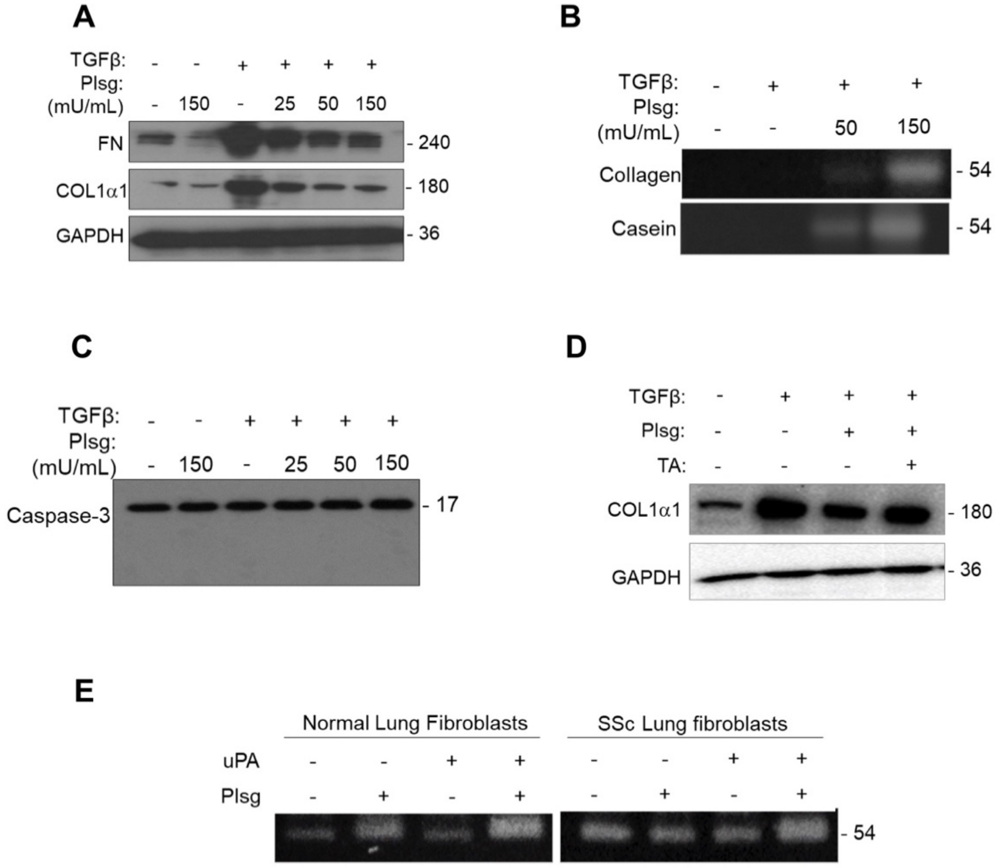

Supplemental Figure 4. Plasminogen exerts anti-fibrotic activity via downregulation of PAI-1 and upregulation of MMP-1 and MMP-3 in vitro. Normal lung fibroblasts were treated with TGF-β1 (10 ng/ml) in combination with increasing concentrations of plasminogen for 72 hours. (A) Protein levels of FN and COL1α1 were analyzed by immunoblotting of lysates. GAPDH was used as a loading control. (B) Fibroblast culture supernatants from (A) were used for detection of MMP activity. Substrates used in zymography are collagen type I (for MMP-1 activity) and casein (for MMP-3 activity). (C) Protein levels of Caspase-3 were analyzed by immunoblotting of fibroblast lysates treated as in (A). (D) Normal lung fibroblasts were treated with TGF-β1 in combination with plasminogen and tranexamic acid for 72 hours. Protein levels of COL1α1 were analyzed by immunoblotting of lysates. GAPDH was used as a loading control. (E) Normal lung fibroblasts and SSc lung fibroblasts were treated with plasminogen in combination with uPA. Fibroblast culture supernatants were used for the detection of MMP activity by zymography using collagen type I as substrate (for MMP-1 activity). Plsg = plasminogen. TA = tranexamic acid.

A

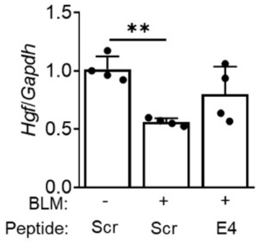

B

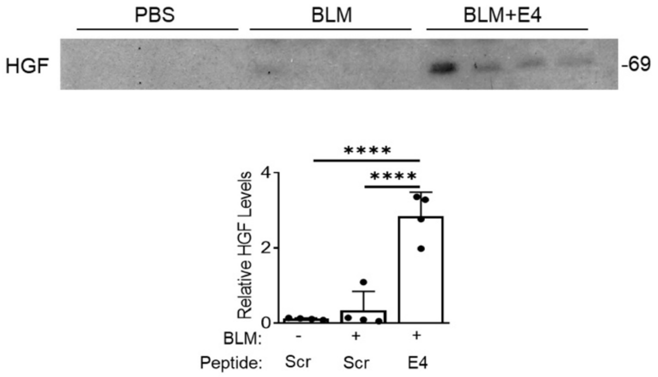

Supplemental Figure 5. E4 increases HGF levels and activity in mouse lung tissues and BAL fluid. Bleomycin (N=4), Bleomycin in combination with E4 (N=4) or PBS (N=4) was administered intratracheally to 6 to 8-week-old C57BL/6J male mice. (A) Lungs were harvested on day 5 post treatment. Expression levels of *Hgf* gene were measured and shown relative to the levels of the housekeeping gene *Gapdh*. (B) BAL fluid was collected on days 7 post treatment and subjected to immunoblotting for HGF detection. The immunoblot (upper) and graphical presentation of the data (lower) are shown. Statistical analysis was performed using One-way ANOVA. $^{**}p < 0.05$, **** $p < 0.0001$. Error bars are mean +/- SD.

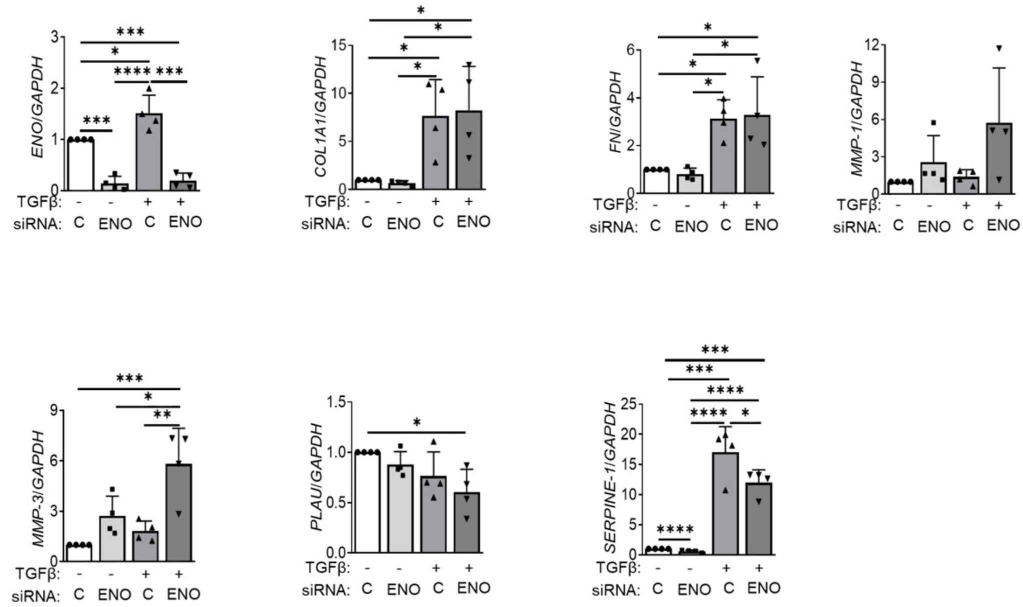

Supplemental Figure 6. Enolase-1 mediates the pro-fibrotic effects of TGF-β1. Normal lung fibroblasts were transfected with control or *Eno-1* siRNA, and then treated with TGF-β1 (10 ng/ml) for 72 hours. mRNA expression levels of *ENO*, *COL1A1*, *FN*, *MMP-1*, *MMP-3*, *PLAU* and *SERPINE-1* were measured relative to the housekeeping gene *GAPDH*. C = Control *si*RNA, ENO = Enolase-1 *si*RNA. Statistical analysis was performed using One-way ANOVA. $*p < 0.05$, $** p < 0.01$, $*** p < 0.001$, $**** p < 0.0001$. Error bars are mean +/- SD.

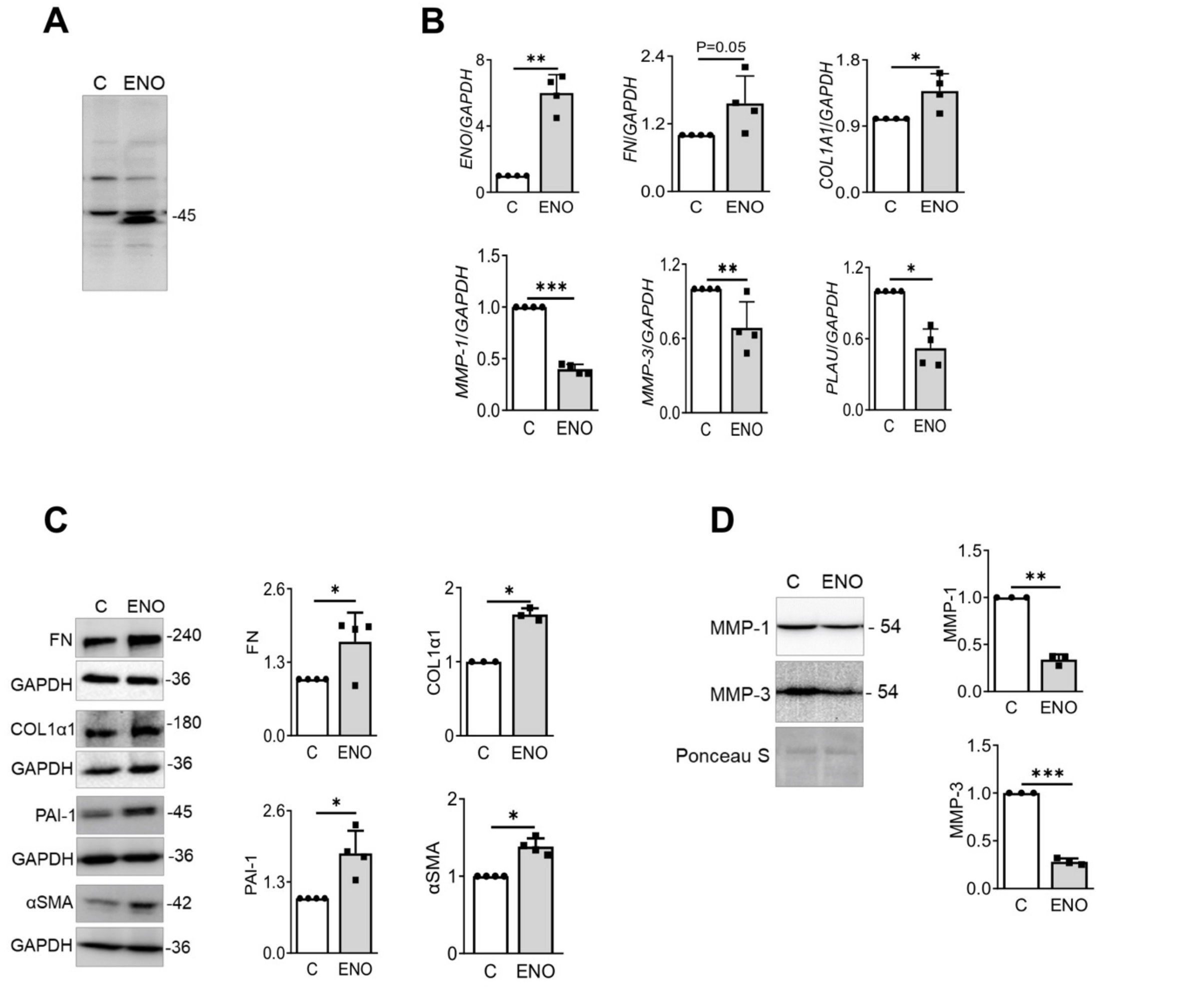

Supplemental Figure 7. Expression of plasmid encoded Enolase-1 in primary normal lung fibroblasts promotes a fibrotic phenotype. Normal lung fibroblasts were transfected with empty vector plasmid (C) or ENO-encoding plasmid for 48-96 hours. (A) ENO was detected by immunoblotting of whole cell lysate with antibody to the V5 tag. Representative western blot showing ENO levels 72 hours post transfection. Lane 1-Empty vector (C); Lane 2- ENO-encoding plasmid (ENO). (B) mRNA expression levels of *ENO*, *COL1A1*, *MMP-1*, *MMP-3*, and *PLAU* were measured 48 hours post transfection and are shown relative to the housekeeping gene *GAPDH.* (C) Protein levels of FN, COL1α1, PAI-1 and αSMA in whole cell lysates were detected by immunoblotting 72 hours post transfection with ENO-encoding plasmid or control plasmid. GAPDH was used as a loading control. A representative immunoblot (Left) and graphical presentation of the data (Right) are shown. (D) Protein levels of MMP-1 and MMP-3 in culture media supernatants of fibroblasts treated as in (C) for 96 hrs were detected by immunoblotting. Ponceau S stain is used to normalize the signal. Statistical analysis was performed using paired Student's t-test *$p < 0.05$, ** $p < 0.01$, *** $p < 0.001$. Error bars are mean +/- SD.
